# Mapping a lateralization gradient within the ventral stream for auditory speech perception

**DOI:** 10.3389/fnhum.2013.00629

**Published:** 2013-10-02

**Authors:** Karsten Specht

**Affiliations:** ^1^Department of Biological and Medical Psychology, University of BergenBergen, Norway; ^2^Department for Medical Engineering, Haukeland University HospitalBergen, Norway

**Keywords:** ventral stream, fMRI, speech perception, auditory perception, temporal lobe

## Abstract

Recent models on speech perception propose a dual-stream processing network, with a dorsal stream, extending from the posterior temporal lobe of the left hemisphere through inferior parietal areas into the left inferior frontal gyrus, and a ventral stream that is assumed to originate in the primary auditory cortex in the upper posterior part of the temporal lobe and to extend toward the anterior part of the temporal lobe, where it may connect to the ventral part of the inferior frontal gyrus. This article describes and reviews the results from a series of complementary functional magnetic resonance imaging studies that aimed to trace the hierarchical processing network for speech comprehension within the left and right hemisphere with a particular focus on the temporal lobe and the ventral stream. As hypothesized, the results demonstrate a bilateral involvement of the temporal lobes in the processing of speech signals. However, an increasing leftward asymmetry was detected from auditory–phonetic to lexico-semantic processing and along the posterior–anterior axis, thus forming a “lateralization” gradient. This increasing leftward lateralization was particularly evident for the left superior temporal sulcus and more anterior parts of the temporal lobe.

## INTRODUCTION

The research on speech perception, language, and human communication behavior has a long history in science and reveals to be an actual topic through centuries and, with the venue of neuroimaging methods, became an even broader research field over the last two decades (Price, 2012). The first important contributions to our current view on the neuroanatomy of language came from the French physician, anatomist, and anthropologist Pierre Paul Broca (1824–1880) and the German physician, anatomist, psychiatrist, and neuropathologist Carl Wernicke (1848–1905). Broca was the first to describe an association between language deficit and the damage of a specific frontal brain area, which is now referred to as “Broca’s area” (Dronkers et al., 2007), while Carl Wernicke noticed that also lesions of the posterior part of the left superior temporal gyrus (STG) could cause language disorders, even though these disorders substantially differed from those deficits caused by frontal lesions (Wernicke, 1874). In a review published in 1885, Lichtheim developed a model of aphasia, proposing the posterior area of the temporal lobe to be involved in the comprehension of language, and the anterior area of the temporal lobe in its expression and production, while an anatomically less defined area was thought to process concepts (Lichtheim, 1885). Thereby, this early model was able to allocate various forms of lesion-induced aphasia to one of these areas, or to damaged connections between them. This model from the end of the nineteenth century was mainly based on clinical observations and neuroanatomical examinations. The majority of later neurological models of language processing focused on the arcuate fasciculus as the dominating fiber tract (Ueno et al., 2011; Weiller et al., 2011). With the venue of functional *in vivo* measurements, such as electrophysiological and imaging techniques, this view has been revised, and the most recent models on speech perception propose a dual-stream processing network (Hickok and Poeppel, 2004, 2007; Scott and Wise, 2004), with a dorsal stream, comparable to the classical language network, and an additional ventral stream. The dorsal stream extends from the posterior temporal lobe of the left hemisphere through inferior parietal areas into the left inferior frontal gyrus, also including premotor areas. Anatomically, this hypothesized stream mainly follows the arcuate fasciculus, connecting the temporal and inferior parietal lobe with the inferior frontal gyrus, and possesses three distinct branches in the left hemisphere (Catani et al., 2007). The second stream is the ventral stream, which is assumed to originate in the upper posterior part of the temporal lobe and to extend toward the anterior part of the temporal lobe, where it also connects to the ventral part of the inferior frontal gyrus through the uncinate fasciculus and extreme capsule (Saur et al., 2008; Weiller et al., 2011). Confirming evidence for this dual-stream perspective come from several neuroimaging studies, presented in a recent review by Price (2012) that summarizes the attempts over the last 20 years in mapping speech perception processes using different neuroimaging methods and paradigms. Furthermore, neurocomputational models deliver further evidence for the dual pathway model, with a dorsal pathway that maps sounds-to-motor programs and is thus important for repetition, and a ventral pathway that is important for the extraction of meaning (Ueno et al., 2011).

Building on the work above, this article describes and reviews the results from a series of complementary functional magnetic resonance imaging (fMRI) and positron emission tomography (PET) studies that aimed to trace the hierarchical processing network for speech comprehension within the left and right hemisphere, with a particular focus on the temporal lobe and the ventral stream. To achieve this goal, the work presented here starts with studies exploring pure auditory processing within the primary and secondary auditory cortex, continues with studies on the processing of vowels and consonants and concludes with studies on the perception of syllables and the processing of lexical, semantic, and sentence information. These processes are the core processes for decoding speech and extracting its meaning and are thus important for communicative abilities. These functions are assumed to be subserved by the ventral stream. Thus, the ventral stream is an important part within the speech and language network as it is involved in both perception and production of speech.

However, there is a specific challenge in exploring auditory and in particular speech perception. Unlike visual information, auditory information is stretched over time and spectro-temporal characteristics are the information carriers. Based on the resonance frequencies of the vocal tract, characteristic patterns emerge that are important in identifying a sound as a speech sound. Various parameters play together. For example, a vowel, e.g., an /a/, is dominated by constant intonation and constant pitch of the voice. By contrast, an unvoiced stop consonant is dominated by a sound produced by the sudden stop of airflow within the vocal tract, and it is characterized by its place of articulation and the voice onset time (VOT; Benkï, 2001). Depending on the configuration of the vocal tract, this results in a very characteristic sound – or noise burst – for a stop consonant, e.g., a /t/. Similarly, the voiced consonant /d/ has a very similar configuration of the vocal tract, with respect to placement of the tongue, opening of the mouth, etc. However, a /d/ does not have an acoustically similar prominent stop of airflow as the /t/, but an earlier insertion of the voice in case of a following vowel, thus making it possible to differentiate a /da/ from a /ta/. Thus, these two syllables share the same place of articulation, but differ in their VOT. A similar association can be found for the syllable pairs /ba/ and /pa/ and /ga/ and /ka/. These described differences between, for example, the consonant–vowel (CV) syllables /da/ and /ta/ are easily visible in spectrograms. It is not only the spectro-temporal difference between, for example, a stop-consonant and a vowel that is characteristic for a speech sound, but also the temporo-spectral sub-structure, called “the formants.” All voiced speech sounds are characterized by these formants, which are resonance frequencies of the vocal tract. In the spectrogram, the formants appear as distinguishable sub-structures in the lower part of the spectrogram and are the same for /da/ and /ta/. Since those CV syllables are important building blocks in several languages, they are often used to study basic speech perception processes, for example in dichotic listening tasks (Rimol et al., 2006a; Sandmann et al., 2007; Hugdahl et al., 2009). Therefore, all or some of the six CV syllables /ba/, /da/, /ga/, /ka/, /pa/, and /ta/ are used as test stimuli in some of the studies presented here (Rimol et al., 2005; van den Noort et al., 2008; Specht et al., 2009; Osnes et al., 2011b).

## MAPPING THE VENTRAL STREAM

The following section describes a series of complementary studies that aimed to disentangle the different processes and neuronal correlates involved in auditory speech perception. The section starts with studies on the basic auditory perception of phonetic signals, such as vowels and consonants, and proceeds to studies on sub-lexical, lexical as well as semantic processing. These processes describe the function of the hypothesized ventral stream that is predominantly mediated through sub-structures of the temporal lobes. The aim of these studies was not only to identify the different processes associated with the ventral stream and to map them onto respective brain areas, but also to map the sensitivity of the contributing brain structures to the presence of phonetic information and to detect on which level a functional asymmetry between brain hemispheres emerges. To achieve this goal, three of the studies presented here were performed using a “dynamic” paradigm (in the following called “sound morphing”; Specht et al., 2005, 2009; Osnes et al., 2011a,b), which is a different experimental setup than typically applied in fMRI studies. Studies on auditory perception often compare categories of stimuli, such as noise, music, or speech (see, e.g., Specht and Reul, 2003). However, in order to assess whether a brain structure responds uniformly to a sound, or whether it is sensitive to the presence of relevant phonetic features, dynamic paradigms have the advantage that they can keep some general acoustic properties constant while varying others. Thus, it is possible to differentiate brain areas that show constant responses from areas that change following the manipulation, as seen, for example, in a study that gradually “morphs” a sound from white noise into a speech sound (Specht et al., 2009; Osnes et al., 2011a). Similar approaches have also been applied earlier by using, for example, noise-vocoded speech (see, for example, Davis and Johnsrude, 2003), where the manipulated sounds originate from undistorted sounds, or by using a morphing procedure for probing categorical perception (Rogers and Davis, 2009). Some of the studies presented here used a similar approach by morphing sounds across sound categories, e.g., from a non-verbal white noise into a speech sound, or from a flute sound into a vowel. These sound-morphing approaches provide additional information on perception processes, as they allow to differentiate between brain areas that follow the manipulation from those that response uniformly to the presence of a sound. Technically, a set of stimuli is generated where the presence of a respective acoustic feature is varied in its presence or intensity. Played in the corrected order, the respective feature becomes more and more audible. In this respect, it is important, that the subjects are naïve to this manipulation and that the sounds are not presented in the correct, gradual order, but randomly, since top-down and expectancy effects are known to influence the perception of distorted or unintelligible sounds (Dufor et al., 2007; Osnes et al., 2012).

**FIGURE 1:**
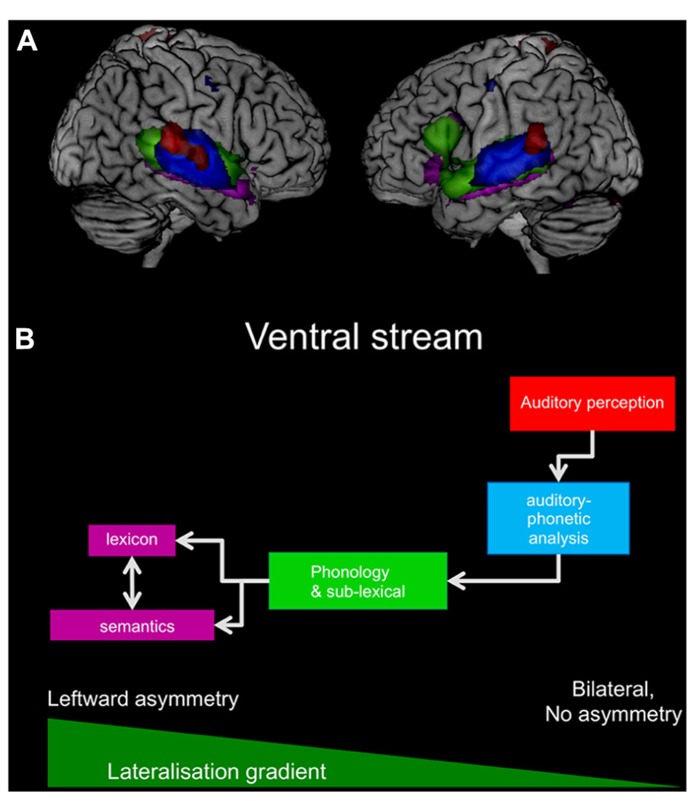
**(A)** The figure summarizes the results from the presented studies and displays the results for auditory processes of vowels in red, auditory phonetic analysis of consonants in blue, phonological, and sub-lexical processes in green, and, finally, lexico-semantic processes in purple. For display purposes are all results converted into *z*-scales and projected onto a standard brain. **(B)** The simplified working model for the ventral stream is displayed with the same colors as in **(A)**. In addition a lateralization gradient indicates the increasing leftward asymmetry.

The studies described below follow a simplified model of the ventral stream, as depicted in **Figure 1**, starting with the auditory–phonetic analysis of vowels and consonants, continuing to sub-lexical, lexical, and semantic processing. It should further be noted that in most studies, if not indicated otherwise, participants performed an attentive, but otherwise passive listening task, with either no task (Specht and Reul, 2003) or an arbitrary task not related to the content of the study (Rimol et al., 2005; Specht et al., 2009; Osnes et al., 2011a,b).

### AUDITORY–PHONETIC ANALYSIS

It has been shown that non-verbal material, including pure tones and complex sounds, elicit asymmetric BOLD signals between the brain hemispheres, with stronger signals in the right posterior part of the STG and right Heschl’s gyrus, while the perception of speech elicits stronger responses on the left (Specht and Reul, 2003). But what happens when the differentiation between verbal and non-verbal content is not that clear, especially when the participant does not recognize a difference between them? This was the central question of the study by Osnes et al. (2011b), where the sound from a flute was gradually changed into either the sound of trumpet or oboe, or alternatively into a vowel /a/ or /o/. This was achieved by a sound-morphing paradigm, where the vowel spectrum was linearly interpolated into the flute spectrum, resulting in a stepwise transition from a flute into a vowel sound over seven distinct steps. Step one was a sound consisting of mainly flute-sound features, while the presence of vowel-sound features increased over the subsequent steps two to seven. Non-phonetic control sounds were created in a similar manner, resulting in a step-wise transition from flute into either an oboe or trumpet sound. It is important to note that participants were not informed about this manipulation and also – after hearing the sounds – were not aware of that the sounds contained phonetic features to a varying degree, as revealed by post-study interviews. This is an important and fundamental study concept that was also used in some of the following studies in order to reduce the effect of expectancy, since the expectance of hearing a speech sound can substantially change the way that the sounds are perceived. This was, for example, impressively displayed in the study by Dufor et al. (2007) and recently replicated by Osnes et al. (2012) using the same stimuli described above. In addition, the level of attention can influence the extent of activation in primary and sensory areas (Jäncke et al., 1999; Hall et al., 2000; Hugdahl et al., 2000), influencing also the within-subject reliability of the activation, as shown for the visual cortex (Specht et al., 2003b). Hence, participants were given an arbitrary task, which was unrelated to the true aim of the study and, more important, did not contain any discrimination between the different sounds. Thus, the results reflect particularly the bottom-up, stimulus-driven brain response and allow to test whether the brain is able to differentiate between such ambiguous sounds that only vary in the degree of phonetic information without being obvious speech sounds. High sensitivity to the used phonetic manipulation was expected in the primary and secondary auditory cortex. The results broadly confirmed this *a priori* hypothesis by demonstrating a clear differentiation between sounds with increasing phonetic information versus sounds with unaltered phonetic information. Especially the STG and planum temporale followed this manipulation logarithmically, while more medial areas, i.e., the core area of the auditory cortex, did not respond to the manipulation. This indicated that the BOLD response prominently increased already in the early phase of the sound-morphing sequence, when only little phonetic information was present, while increases in the BOLD response were less prominent in the later phase of the morphing sequence. In addition, no obvious lateralization effects were observed, indicating that left and right posterior temporal lobes were equally sensitive to this manipulation (Osnes et al., 2011b).

Stop-consonants are even more important building blocks of speech than vowels. As described above, stop consonants are consonants in which the sound is produced by stopping the airflow in the vocal tract either with or without simultaneous voicing (voiced/unvoiced consonant), thus containing rapid frequency modulations. Rimol et al. (2005) explored the neuronal responses to unvoiced stop consonants. The results demonstrated bilateral activations in the temporal lobes with a clear leftward asymmetry for both consonants as well as CV syllables. This leftward asymmetry was further confirmed by direct comparison with the matched noise condition. A leftward asymmetry for consonants as opposed to vowels (Osnes et al., 2011b) could indicate a higher temporal resolution of the left primary and secondary auditory cortex (Zatorre and Belin, 2001; Zatorre et al., 2002; Boemio et al., 2005), which is then further reflected in a general left dominant processing of those speech-specific signals. This may to a certain degree confirm the asymmetric sampling theory (AST; Poeppel, 2003), although the left–right dichotomy in temporal resolution may oversimplify the underlying processes (McGettigan and Scott, 2012).

Nevertheless, the results of these studies clearly indicate that the different sound structures of consonants and vowels, with rapid frequency modulations for stop consonants and a more constant tonal characteristic for vowels, are differently processed by the two temporal lobes. More specifically, the left temporal lobe clearly has a higher sensitivity for consonants, while vowels are processed more bilaterally. This result was also confirmed by a study that used a dichotic presentation of CV syllables, where the functional asymmetry was explored on a voxel-by-voxel level (van den Noort et al., 2008). Besides bilateral activations, the results indicated a functional asymmetry toward the left, with significantly higher activations in the left posterior STG, extending into the angular and supramarginal gyrus.

Interestingly, these results are paralleled by behavioral investigations of the VOT effects in the dichotic listening task. In such a task, two CV syllables are presented to the participant at the same time, and the participant has the task to repeat the syllable that is perceived the most clearly. In most of the cases, this will be the syllable that was presented to the right ear (Hugdahl et al., 2009; Hugdahl, 2011), an effect termed “right ear advantage” (REA). However, the strength of the REA depends on the VOT. The strongest REA was observed when a syllable with a long VOT was presented to the right ear (Rimol et al., 2006a; Sandmann et al., 2007). These are also those syllables with the most complex temporo-spectral characteristics, thus likely benefiting from the assumed higher temporal resolution of the left auditory cortex (Zatorre et al., 2002), since signals from the right ear are predominantly transmitted to the left auditory cortex.

### SUB-LEXICAL PROCESSING

In order to explore the phonological and sub-lexical decoding in more detail, the following study used again the sound-morphing procedure to investigate the dynamic of the responses in the posterior and middle part of the STG. This was achieved by sound-morphing white noise, i.e., a sound with equal spectral and temporal distribution, in seven distinct steps (“Step 1” to “Step 7”) into either a speech sound or a short music sound. The latter served as control stimuli. In order to have a comparable spectral complexity of the target sounds, the sounds were selected based on their spectral characteristics. The speech sounds were the CV syllables /da/ and /ta/, and the music instrument sounds were a piano chord consisting of a major triad on a C_3_ root, and an A_3_ guitar tone (see Specht et al., 2009 for technical details). It is important to note that the stimuli were presented in a randomized order, i.e., that the participants never heard the stimuli in a sequential order from Step 1 to Step 7 to avoid expectation effects, as explained previously. As before, the participants performed an arbitrary task and were debriefed about the real aims of the study afterward. Parallel behavioral assessment was conducted in an independent sample of subjects to ensure that the subjects were naïve to the stimulus material in both studies (Osnes et al., 2011a).

While the previously described studies on auditory–phonetic processing revealed a high sensitivity of the STG to phonetic cues and demonstrated no lateralization for vowels, but a clear lateralization for stop consonants and CV syllables, the results of this study bridges the previous results by demonstrating an increasing lateralization toward the left as the sound became more and more a speech sound (CV syllable). Moreover, this increased leftward asymmetry was particularly prominent outside of the auditory cortex. More precisely, there was a small area in the middle part of the left superior temporal sulcus (mid-STS) that showed the strongest differentiation between the sounds along with a significant interaction between speech and music sound manipulations, and increasing response and increasing leftward asymmetry with increasing intelligibility of the speech sounds was demonstrated. Furthermore, this area (MNI coordinates -54, -18, -6) overlaps with the mid-STS area (MNI coordinates -59, -12, -6) that was detected in an earlier study that compared the perception of real words with complex sounds and pure tones (Specht and Reul, 2003). In contrast, when the sound morphed into a music sound, no lateralization was found, and activity in left and right temporal lobe areas increased to a comparable extent. In addition, a parallel behavioral study in a naïve sample of participants demonstrated that the participants were more able to identify distorted speech sounds as speech than the distorted music sounds as music (Osnes et al., 2011a). Interestingly, at an intermediate step, the breaking point from where on subjects perceived the sounds as speech sounds, there was additional activation in the premotor cortex, possibly indicating processes that facilitate the decoding of the perceived sounds as speech sounds. This link between speech perception processes and areas belonging to the dorsal-stream have been described before in case of degraded speech signals (Scott et al., 2009; Peelle et al., 2010; Price, 2010, 2012). Using dynamic causal modeling (DCM), Osnes et al. (2011a) was able to demonstrate that the connection between the premotor cortex and STS was bidirectional, while the connection from the planum temporale to the premotor cortex was only one-directional (forward), possibly reflecting a directed flow of information. Note that the premotor cortex was only involved when the sound was morphed into a speech sound, but that there were no connections between premotor cortex and STS or planum temporale, when the sound was morphed into a non-verbal sound.

It is important to emphasize that activations were always seen in both temporal lobes irrespective of the presented sound, but that only the left STS demonstrated an additional sensitivity to the sound-morphing manipulation. This, however, indicates only a higher sensitivity to the manipulation, but not necessarily a speech-specific activation.

Furthermore, there was no observable lateralization or exclusive processing of one stimulus category over the other on the level of the primary and secondary auditory cortex. This lack of lateralization in primary auditory processing is especially present in attentive but otherwise passive listening studies, while lateralization (leftward asymmetry) was observed in syllable discrimination tasks (Poeppel et al., 1996). Once a signal is identified as a speech stimulus, a stronger leftward asymmetry might emerge, indicating further phonetic and phonological processing (Specht et al., 2005). However, it is still an open question whether the identification of an acoustic input as speech sound is a bottom-up and thus stimulus-driven effect, or a top-down process. The results presented here indicate, at least to a certain extent, a bottom-up effect.

### LEXICAL PROCESSING

In a third study that used the sound-morphing paradigm, only real words were used, but filtered in such a way that the sounds were identifiable as speech while at the same time varied in the degree of intelligibility (Specht et al., 2005). The results confirmed that especially the left temporal lobe is sensitive to the intelligibility of a speech sound, while the right temporal lobe responds in a comparable way to all stimuli, irrespective of the sound category. This was seen in both the voxel-wise analysis as well as region-of-interest analysis with a priori defined regions in the left and right temporal and frontal lobes. Note that once again the right temporal lobe responded to all stimuli, but did not follow the manipulation, in contrast to the left hemisphere. The increasing intelligibility of the words was also reflected by an increased activity within the left inferior frontal gyrus, comprising the dorsal-posterior part of Broca’s area [Brodmann area (BA) 44], which may be due to an active processing of the distorted sounds, as subjects had to indicate by button press when the sound was intelligible, and may thus reflect a lexical processing of the stimuli.

These lexical processes were further explored by a lexical decision task, in which participants were asked to perform a decision between, either real words and phonologically incorrect non-words, or, as a more demanding task, between real words and phonologically correct but otherwise meaningless pseudo-words. A high-low pitch decision served in both cases as auditory control condition. The results from this PET study demonstrated that the easier non-word/real word decision was made by a phonological analysis, involving only on the temporal lobe, in particular left temporal structures, without any involvement of frontal areas. By contrast, the more demanding pseudo-word decision involved also the left inferior frontal gyrus, including Broca’s area (BA 44, 45), which is also in line with other studies on lexical decision making that use, for example, visual presentations (Heim et al., 2007, 2009).

### SEMANTIC PROCESSING

The last process examined by the study series described here was semantic processing, a processing step distinct from lexical processing. In order to get these processes separated in the imaging data and to separate them also from auditory–phonetic processing, the respective study by Specht et al. (2008) used an independent component analysis (ICA; Calhoun et al., 2005, 2009; Keck et al., 2005; Kohler et al., 2008) rather than a univariate general linear model approach. The paradigm comprised three different linguistic levels. The first level was a passive perception of reversed words, which was used to control for auditory perception and, partially, for phonological processing. The second level was a passive listening to real words, which aimed to control for phonological and lexical processing. Finally, the third level was a covert naming task after aurally presented definitions, that reflects in particular semantic processing, but may to a certain degree be confounded by sentence processing. Hence, all three levels were expected to activate different processing stages of the ventral stream – or “what pathway” (Scott and Wise, 2004) – to different degrees.

An ICA is beneficial here as it has the ability to combine the involved brain areas to networks that show the same time course in the BOLD signal and share the same variance. Since the auditory and phonological processing was present in all three levels, the ICA was able to separate the respective network from the network for semantic and sentence processing that was only required in the naming task.

The two main components that were detected by the ICA, confirmed that the auditory processing of phonological information is an almost bilateral process, while speech comprehension, comprising lexical and semantic processing, is often left lateralized (Hickok and Poeppel, 2004, 2007; Poeppel et al., 2012). In particular, the left anterior temporal lobe (ATL) has been identified as an important structure for required for semantic and naming tasks (Schwartz et al., 2009; Binder et al., 2011). The areas of the second ICA component also nicely overlap with the ventral stream model, including mainly anterior portions of the temporal lobe, but also the temporo-parietal junction and a distinct area in the posterior part of the inferior temporal gyrus (ITG; Specht et al., 2008). An extension from the posterior superior temporal areas toward the temporal pole, forming the ventral stream, is a typical finding (Scott et al., 2000). This posterior–anterior extension reflects that the more the complexity of linguistic processing increases by involvement of semantic processing and sentence comprehension, the greater becomes the extension of activation to anterior and ventral parts of the temporal lobe. Also involved are inferior, posterior areas including the ITG, as repeatedly reported in studies on sentence processing and semantic aspects of language (Rodd et al., 2005; Humphries et al., 2006, 2007; Hickok and Poeppel, 2007; Patterson et al., 2007; Binder and Desai, 2011; Poeppel et al., 2012).

Interestingly, a very similar pattern is often found when analyzing the loss of gray matter in patients suffering from primary progressive aphasia (PPA), which is an aphasic syndrome caused by neuronal degeneration that can occur in different clinical variants (Grossman, 2002; Mesulam et al., 2009; Gorno-Tempini et al., 2011). Its neuropsychological syndrome is characterized by slowly progressing isolated language impairment without initial clinical evidence of cognitive deficits in other domains (Grossman, 2002; Mesulam et al., 2009). In particular, the clinical phenotype of semantic dementia, which may be a variant of a fluent PPA (Adlam et al., 2006), is mainly associated with damage to the temporal lobe, with the left ATL being affected most severely with respect to gray matter atrophy (Mummery et al., 2000; Adlam et al., 2006; Mesulam et al., 2009) and white matter damage (Galantucci et al., 2011). Although less common and less pronounced, ATL pathologies, in combination with parietal lobe pathologies, have also been observed in the non-fluent, logopenic PPA sub-type, as well (Zahn et al., 2005).

All results from the studies presented here are summarized in **Figure 1**. The summary depicts the ventral stream and displays in particular how the activation extends from the primary auditory cortex to anterior parts of the temporal lobe as the perceived sound becomes a meaningful speech stimulus, a real word, or a sentence. Furthermore, **Figure 1** indicates that the ventral stream is bilateral, but more extended on the left hemisphere. Only the left inferior frontal gyrus demonstrates a significant contribution to the processing.

## DISCUSSION

Auditory speech perception is, as illustrated in this summary, a complex interaction of different brain areas that are integrated into a hierarchical network structure. To unravel the neuronal mechanisms of speech perception, it is of crucial importance to follow and to understand the organization of the information flow, particularly within the temporal lobes. Although auditory perception has been investigated by numerous functional imaging studies over the last decades, several aspects are still unresolved and not fully understood. One important contribution to the description of the processes behind auditory speech perception was the introduction of the concepts of the dorsal and ventral streams in recent models of speech perception (Hickok and Poeppel, 2004, 2007; Scott and Wise, 2004). On the neuroanatomical level, these two processing streams can to a certain degree be linked to two fiber tracts and their sub-branches (Catani et al., 2004, 2007; Saur et al., 2008; Weiller et al., 2011). However, one has to bear in mind that those theoretical concepts of “streams” do not necessarily have to follow neuroanatomical structures. Although this concept of two processing streams is striking, it is difficult to display them with functional neuroimaging, since neuroimaging results typically provide “snapshots” of brain activations rather than dynamic processes. Therefore, the series of complementary studies presented above focused particularly on two aspects: first, to create a series of studies that overlapped with respect to mapping the different processing nodes within the hierarchical network that configures the ventral stream in the temporal lobe, and, second, to use dynamic paradigms in which stimulus properties were gradually changed in order to identify brain areas that were sensitive to the manipulation. Thereby, speech sensitive areas could be separated from areas of general auditory perception, or lexical from sub-lexical areas.

The studies have consistently shown that speech perception is not a pure left hemispheric function. It is the interplay of the different left and right temporal lobe structures that generates a speech percept out of an acoustical signal, and the left and the right auditory systems process different aspects of the speech signal. Tonal aspects, such as the vowel, do not exhibit a strong lateralization. In contrast, the perception of consonants demonstrates a leftward asymmetry, supporting the hypothesis of different processing capacities and properties of the left and right auditory cortex with respect to temporal and spectral resolution (Zatorre et al., 2002), as well as temporal integration windows, as proposed by the “asymmetric sampling in time” (AST) hypothesis (Poeppel, 2003). However, this simple dichotomy of higher versus lower temporal resolution in the left and right temporal lobe, respectively, may oversimplify the underlying processes as well as the characteristics of speech sounds. Thus, future models should take the specific nature of speech sounds into account, given by the flexibility and limitations of the articulatory system that produces these sounds (McGettigan and Scott, 2012). Nevertheless, the differential processing within the left and right temporal lobe becomes in particular evident when comparing the study that used only vowels (Osnes et al., 2011b) to the study that focused on the processing of stop-consonants (Rimol et al., 2005) or dichotically presented CV syllables (van den Noort et al., 2008; Specht et al., 2009). While the more tonal vowels did not exhibit a left–right asymmetry, consonants and CV syllables were processed stronger by the left than the right auditory cortex and surrounding areas. Note that only asymmetries were detected on this level, but not clear unilateral processes. It is further important to note that the area of the planum temporale did not turn out to be speech specific, although has also been discussed for a long time as an area important for phonological processing. However, in agreement with recent neuroimaging studies, this view has been challenged, and it has been shown that the area of the planum temporale is also involved in early auditory processing of non-verbal stimuli, spatial hearing, as well as auditory imagery (Binder et al., 1996; Papathanassiou, 2000; Specht and Reul, 2003; Specht et al., 2005; Obleser et al., 2008; Isenberg et al., 2012; Price, 2012).

One area that repeatedly appears in the neuroimaging literature on vocal, phonological, and sub-lexical processing is the STS (Belin et al., 2000; Jäncke et al., 2002; Scott et al., 2009; Price, 2010, 2012). The importance of this structure was also supported by the studies presented here that showed distinct, mainly left-lateralized responses during passive listening to syllables and words, when compared to non-verbal sounds within the middle part of STS (Specht and Reul, 2003; Specht et al., 2005, 2009). However, it should again be emphasized that the results only indicate a high sensitivity to the phonological signals and a high sensitivity to sound-morphing manipulations, without necessarily implying that this is a speech-specific area. It is possible that a speech-specific involvement of the STS may emerge when required (Price et al., 2005). Interestingly, when the focus is on phonological processing, the left STS appears to be the dominating structure, while when voice aspects are in the focus, the right STS is more dominant (Belin, 2006; Latinus and Belin, 2011). Moreover, a recent meta-analysis by Hein and Knight indicated that the STS of the left and right hemisphere is apparently involved in several different processes involving not only phonological processing, but also theory of mind, audio-visual integration, or face perception (Hein and Knight, 2008). Thus, studies are required that examine these function on a within-subject level in order to verify the neuroanatomical overlap of these different functions. Besides the areas in the STG and STS, several studies also pinpoint an area in the posterior part of the ITG, close to the border to the fusiform gyrus. This area is typically seen in visual lexical decision task (see, for example, Heim et al., 2009), but also in auditory tasks, such as word and sentence comprehension (Rimol et al., 2006b; Specht et al., 2008). In general, there is reasonable evidence that this area serves as a supramodal device in which the auditory and the visual ventral streams meet or join. Thus, this area is independent from the input modality and has to be differentiated from an adjacent area, often referred to as the “visual word form area,” which is located more posterior and medial (Cohen et al., 2004). The function of this inferior temporal area is still under debate, but several studies point to the fact that this area is especially involved in lexical processing. In accordance with that, the model by Hickok and Poeppel (2007) calls this area the “lexical interface.” Interestingly, the same or nearby areas seem also to play an important role in multilingualism (Vingerhoets et al., 2003) and show also structural and functional alterations in subjects with dyslexia (Silani, 2005; Dufor et al., 2007).

Moving further along the ventral stream toward the anterior portion of the temporal lobe, the neuroimaging results presented here demonstrate, in agreement with the literature (Vandenberghe et al., 2002; Price, 2010, 2012; Binder and Desai, 2011), an increasing contribution of more anterior portions of the temporal lobe to lexical, semantic, and sentence processing (Specht et al., 2003a, 2008). This shift from acoustic and phonological processing in the posterior superior temporal lobe to semantic processing in the ATL characterizes the ventral stream (Scott et al., 2000; Visser and Lambon Ralph, 2011). Interestingly, neurocomputational models confirm this gradual shift within the ventral stream. Ueno et al. (2011) modeled a neuroanatomically constrained dual-stream model, with a dorsal and a ventral stream. They were able to demonstrate the division of function between the two streams, and they were also able to demonstrate that a gradual shift from acoustic to semantic processing along the ventral stream improves the performance of the model (Ueno et al., 2011). However, this model was constrained to an intra-hemispheric network with one ventral and one dorsal stream only and did not consider any functional asymmetry. In contrast, neuroimaging data indicate a bilateral representation of some parts of the ventral stream (Hickok and Poeppel, 2007). This is reflected by different degrees of functional asymmetries along the ventral stream. While auditory and sub-lexical processing are more symmetrically organized, a stronger leftward asymmetry appears for lexical and semantic processes, which is in line with the notion that a leftward asymmetry for linguistic processes emerges only outside of the auditory cortex and adjacent areas (Binder et al., 1996; Poeppel, 2003). Furthermore, there is emerging evidence that semantic processing and conceptual knowledge are crucially dependent on the functional integrity of the ATL, including among other areas the left ventrolateral prefrontal cortex and the left posterior temporal and inferior parietal areas. This was demonstrated by, for example, TMS studies (Lambon Ralph et al., 2009; Pobric et al., 2009; Holland and Lambon Ralph, 2010), studies using direct cortical stimulation (Luders et al., 1991; Boatman, 2004), intracranical recording studies (Nobre and McCarthy, 1995), studies in patients with semantic dementia (Patterson et al., 2007; Lambon Ralph et al., 2010), and studies that combined TMS, fMRI, and patient data (Binney et al., 2010). In addition, one has to distinguish between the anterior STG/STS and the ventral ATL that appear to host related but nevertheless distinct functions (Spitsyna et al., 2006; Binney et al., 2010; Visser and Lambon Ralph, 2011). The anterior STG/STS area is considered to be more related to the semantic and conceptual processing of auditory words and environmental sounds, while the ventral ATL is assumed to be a more heteromodal cortical region (Spitsyna et al., 2006). This might indicate a higher level of the ventral ATL within the processing hierarchy, since unimodal visual and auditory language processing streams converge in this heteromodal area (Spitsyna et al., 2006). Furthermore, differential contributions of the left and right ATL have been identified by the demonstration that the left ventral ATL responds stronger to auditory words, while visual stimuli and environmental sounds cause bilateral responses (Visser and Lambon Ralph, 2011). However, it is important to note that particularly the ventral ATL is difficult to access with fMRI, as susceptibility artifacts affect the signal-to-noise ratio in this area. Thus, it is difficult to examine the specific function of this area, and many studies may overlook this structure or are “blind” its responses (Visser et al., 2010, 2012).

Based on the neuroimaging data summarized in **Figure 1**, and in accordance with the literature, a “lateralization gradient” could be proposed for the ventral stream that becomes stronger left lateralized along the posterior–anterior axis (Peelle, 2012). However, this increasing leftward asymmetry, i.e., increasing strength of the lateralization gradient, could also be induced or influenced by top-down control, since a lexical and semantic process implies an active processing of the perceived speech signals rather than simply passive listening. Accordingly, studies that are based on a more passive processing of the speech signals are often showing more bilateral results than studies in which subjects are asked to process the stimuli actively, thus influencing the steepness of the proposed lateralization gradient. Furthermore, the information and stimulus type can influence the steepness of the proposed lateralization gradient, since the strongest lateralization for ATL structures appears for aurally perceived information, such as administered in the studies presented above, but might be less asymmetric for non-verbal, visual information, or figurative language (Binder et al., 2011; Visser and Lambon Ralph, 2011).

In contrast, the observed frontal activations were strictly left lateralized. As depicted in **Figure 1**, the activations extend bilaterally from the primary auditory cortex along the posterior–anterior axis of the temporal lobes, as the sound becomes meaningful speech, with additional involvement of only the left inferior frontal gyrus for lexico-semantic processing. Anatomically, this connection from the anterior portion of the left ATL to the inferior frontal gyrus is most probably provided by a connection via the extreme capsule (Saur et al., 2008; Weiller et al., 2011). However, this inferior frontal contribution is likely to reflect a top-down processing of the stimulus rather than a stimulus-driven bottom-up effect (Crinion et al., 2003), as these activations occurred only in studies using an active task on the lexical and semantic level, and are thus not considered to be a fundamental part of the ventral stream.

In general, it is clear that the temporal lobe, in particular the left temporal lobe, is of crucial importance for speech perception and other language related skills, such as reading and general lexical processing in more posterior and inferior portions of the temporal lobe (Price, 2012). Furthermore, the middle part of the left STS has repeatedly been described as an area central for speech perception. This emphasizes the importance of the ventral stream in the larger speech and language network. The ventral stream, and in particular the ventral stream within the left temporal lobe, is thus important for both perception as well as production of speech. Rauschecker and Scott (2009) proposed a closed loop by incorporation of the dorsal stream into their loop model. As demonstrated here, the ventral stream may terminate in the ATL or, perhaps, in the inferior frontal gyrus. In the later case, this stream has direct connection to the dorsal stream, providing also an anatomical basis for the proposed processing loop. Furthermore, Rauschecker and Scott (2009) proposed a loop for forward mapping and inverse mapping. To some extent, the study by Osnes et al. (2011a), using DCM in combination with the sound-morphing paradigm, demonstrated a link between the dorsal and ventral streams through an involvement of the premotor cortex. The DCM results further demonstrated that the premotor cortex has a bidirectional connection with the STS, but only a forward connection from planum temporale to the premotor cortex, resulting in a directed information flow, similar to the inverse loop proposed by Rauschecker and Scott (2009). Thus, this result helps to understand the perception processes in situations of degraded speech signals. It could also shed some light on disturbed processing networks, as for example found in developmental stuttering, for which there is evidence for functional (Salmelin et al., 1998, 2000) as well as structural (Sommer et al., 2002) alterations of the dorsal stream. In line with this hypothesis, different contributions of the dorsal and ventral stream in speech perception processes has recently been confirmed in a not yet published fMRI study in developmental stutterers (Martinsen et al., unpublished), using the same sound-morphing paradigm as introduced here (Specht et al., 2009).

In summary, the body of data presented here, derived from a series of stepwise overlapping studies that included the use of dynamic paradigms, demonstrates that auditory speech perception rests on a hierarchical network that particularly comprises the posterior–anterior axes of the temporal lobes. It has further been shown that the processes are increasingly leftward lateralized as sounds gradually turn into speech sounds. Still, areas of the right hemisphere are also involved in the processing, which might be beneficial in the case of a stroke. While a multitude of studies demonstrate that temporal lobe structures are essential for speech perception and language processing in general, the fact that the same areas have been shown to be involved in other, non-speech related processes as well, should not be neglected. Thus, new models are needed that can unify and explain such diverging results within a common framework.

## Conflict of Interest Statement

The author declares that the research was conducted in the absence of any commercial or financial relationships that could be construed as a potential conflict of interest.
